# Camrelizumab combined with anlotinib as second-line therapy for metastatic or recurrent small cell lung cancer: a retrospective cohort study

**DOI:** 10.3389/fonc.2024.1391828

**Published:** 2024-07-08

**Authors:** Shujing Shen, Xingya Li, Sanxing Guo, Liang Xu, Ningning Yan

**Affiliations:** ^1^ Department of Radiation Oncology, The First Affiliated Hospital of Zhengzhou University, Zhengzhou, China; ^2^ Department of Medical Oncology, The First Affiliated Hospital of Zhengzhou University, Zhengzhou, China; ^3^ Prevention and Cure Center of Breast Disease, The Third Hospital of Nanchang City, Nanchang, Jiangxi, China

**Keywords:** camrelizumab, anlotinib, small-cell lung cancer, extensive stage, second-line setting

## Abstract

**Introduction:**

This retrospective study evaluates the efficacy of camrelizumab combined with anlotinib versus chemotherapy in patients with extensive-stage small-cell lung cancer (ES-SCLC) undergoing second-line treatment.

**Methods:**

Data were sourced from medical records at a Chinese medical facility, involving 34 patients diagnosed with ES-SCLC after failing first-line treatment. Patients were divided into two groups: one received camrelizumab (200 mg every 3 weeks) with anlotinib (12 mg daily for 14 days followed by a 7-day rest), while the other group received physician-chosen chemotherapy administered every 3 weeks. The primary endpoint was progression-free survival (PFS), with secondary endpoints including overall survival (OS), objective response rate (ORR), and disease control rate (DCR).

**Results:**

The combination therapy group showed a significant improvement in PFS compared to the chemotherapy group (median PFS: 7 months vs. 3 months; hazard ratio (HR): 0.34; 95% confidence interval (CI): 0.15-0.77; p<0.001). However, there was no statistically significant difference in OS between the groups (16.3 months vs. 17.3 months; p=0.82). The ORR was 52.9% in the combination therapy group versus 23.5% in the chemotherapy group (p=0.08), and the DCR was 82.4% compared to 58.8% (p=0.26). Grade 3 or higher adverse events were observed in 17.6% of the combination therapy group and 29.4% of the chemotherapy group.

**Conclusions:**

The findings suggest that the combination of camrelizumab and anlotinib offers a superior anti-tumor response with a manageable safety profile in a second-line setting for ES-SCLC patients. This combination regimen may be a viable option for second-line ES-SCLC treatment.

## 1 Introduction

Small cell lung cancer (SCLC) constitutes approximately 15% of all lung cancer cases and is distinguished by its aggressive growth, high propensity for metastasis, and substantial mortality rate (1, 2). Previous research has indicated that the 5-year survival rate for SCLC patients remains below 5% (3, 4). SCLC is typically classified into two stages: limited stage and extensive stage, as defined by the Veterans Affairs Lung Study Group staging system. Notably, at the time of diagnosis, the majority of SCLC cases are identified as extensive stage SCLC (ES-SCLC) (5), and the prognosis for these patients is generally poor. Recurrence of cancer is common in ES-SCLC patients post-first-line treatment, largely due to the development of drug-resistant tumor cells, despite an initial high sensitivity to first-line therapies (6). Currently, effective second-line treatment options for ES-SCLC are scarce. Topotecan is the standard second-line therapeutic agent, but its objective response rate (ORR) is relatively low, and the significant adverse effects associated with its administration often curtail its clinical utility (7–9). Moreover, prior studies have shown that the overall survival (OS) for patients treated with topotecan is approximately 26 weeks (10).

The advent of immune checkpoint inhibitors (ICIs) has markedly altered the therapeutic paradigm and improved survival outcomes for ES-SCLC patients. The incorporation of the PD-L1 inhibitor atezolizumab into platinum-etoposide chemotherapy as a first-line treatment has significantly extended OS in ES-SCLC patients, establishing it as the preferred first-line treatment regimen (11). Supporting the ICI-based approach, durvalumab, another PD-L1 inhibitor, combined with chemotherapy, has also been endorsed as a first-line treatment for ES-SCLC (12). Furthermore, PD-1 inhibitors such as nivolumab and pembrolizumab have been approved for use in the third-line setting (13, 14). However, it is important to recognize that the efficacy of ICI monotherapy in second-line or subsequent settings is limited to a small subset of unselected ES-SCLC patients, with ORR ranging from 10% to 30%. Thus, there remains a critical need for more effective therapeutic strategies in this clinical context (13, 14).

Combining ICIs with anti-angiogenic agents has emerged as a promising strategy to enhance cancer immunotherapy’s effectiveness (15, 16). Anlotinib, a new multi-target small molecular polytyrosine kinase inhibitor, has shown potential in inhibiting several key receptors, including vascular endothelial growth factor receptor (VEGFR), fibroblast growth factor receptor (FGFR), platelet-derived growth factor receptor α and β (PDGFRα/β), and c-kit (17). This broad inhibition disrupts tumor angiogenesis and growth, contributing to its anti-cancer properties. Camrelizumab, a PD-1 inhibitor, works by blocking the PD-1/PD-L1 pathway, which tumors exploit to evade immune detection. By inhibiting this pathway, camrelizumab reactivates effector T cells, enhancing the body’s immune response against cancer cells (18). Recent studies have indicated that the combination of PD-1 inhibitors with anti-angiogenic drugs can produce synergistic anti-tumor effects. Abnormalities in the tumor microenvironment (TME), driven by VEGF-mediated angiogenesis, contribute to immunosuppression and reduced efficacy of PD-1/PD-L1 blockade (19). VEGF-induced abnormal vasculature in tumors creates hypoxic and acidic conditions, which inhibit effective T cell responses (20). By normalizing the tumor vasculature, anti-angiogenic agents like anlotinib can enhance T cell infiltration and improve the effectiveness of ICIs (21). Given these findings, our study aims to evaluate the effectiveness and safety of combining camrelizumab with anlotinib as a second-line treatment for ES-SCLC, compared to standard chemotherapy regimens.

## 2 Materials and methods

### 2.1 Patients

A retrospective analysis was performed on patients with ES-SCLC who demonstrated disease progression after first-line treatment at the First Affiliated Hospital of Zhengzhou University from October 2017 to September 2019. Eligibility criteria included: patients over 18 years of age with histopathologically or cytologically confirmed ES-SCLC or relapsed SCLC, all of whom had progressed following first-line treatment. Additional criteria were: an Eastern Cooperative Oncology Group (ECOG) Performance Status (PS) of 0 to 1, at least one measurable target lesion as per the Response Evaluation Criteria in Solid Tumors (RECIST) version 1.1, and adequate organ function evidenced by baseline laboratory tests. Key exclusion criteria were: receiving camrelizumab plus anlotinib as a first-line therapy (though other ICIs or VEGF/VEGFR inhibitors were permissible), and the presence of mixed non-small cell lung cancer (NSCLC) elements. Ethical approval for this study was obtained from the Ethics Committee of the First Affiliated Hospital of Zhengzhou University.

### 2.2 Treatment

Patients were divided into two groups: the combination therapy group received camrelizumab with anlotinib, while the chemotherapy group received physician-chosen chemotherapy. The dosages for camrelizumab and anlotinib were based on standard recommendations from their prescribing information. Specifically, camrelizumab was administered at a dose of 200 mg every 3 weeks. Anlotinib was given at a dose of 12 mg daily for 14 days followed by a 7-day rest period. To ensure treatment tolerability, particularly with anlotinib, dose adjustments were made based on individual patient tolerance and comorbidities, such as hypertension. Patients continued to receive treatment until disease progression or the occurrence of intolerable toxicities.

### 2.3 Data collection

Treatment response in patients with ES-SCLC was assessed by the attending oncologist using the RECIST version 1.1, typically conducted every 6 to 8 weeks. Imaging for all patients was independently reviewed by two radiologists. Our analysis encompassed various potential prognostic factors, including sex (male vs. female), age (≥65 years vs. <65 years), smoking history (never smokers vs. former or current smokers), ECOG PS (score of 0-1), clinical stage (IIIB/C vs. IV), presence of brain metastases (yes or no), and receipt of radiation therapy, which could include chest radiation, whole brain radiation, or prophylactic cranial irradiation. The ORR was defined as the sum of complete responses (CR) and partial responses (PR), while the disease control rate (DCR) included CR, PR, and stable disease (SD). Progression-free survival (PFS) was measured from the initiation of treatment until tumor progression, patient death, or the last follow-up date. For survival analysis, patients were censored at their most recent visit if still alive and without disease progression. OS was calculated from the start of treatment to the time of death.

### 2.4 Statistical analysis

All statistical analyses were performed using SPSS version 21.0 (IBM Corp., USA). Continuous or ordinal variables with a normal distribution were reported as the mean ± standard deviation (SD) and analyzed using the independent sample t-test. Variables not normally distributed were described using the median with range and analyzed using the Mann-Whitney U test. Associations between categorical variables were evaluated using the Chi-squared test or Fisher’s exact test, as appropriate. A two-sided p-value of less than 0.05 was considered statistically significant. Kaplan-Meier analysis was employed to calculate PFS, and both univariate and multivariate analyses were conducted to examine the relationships between clinicopathological features and outcomes in terms of PFS.

## 3 Results

### 3.1 Patients

**Figure 1:**
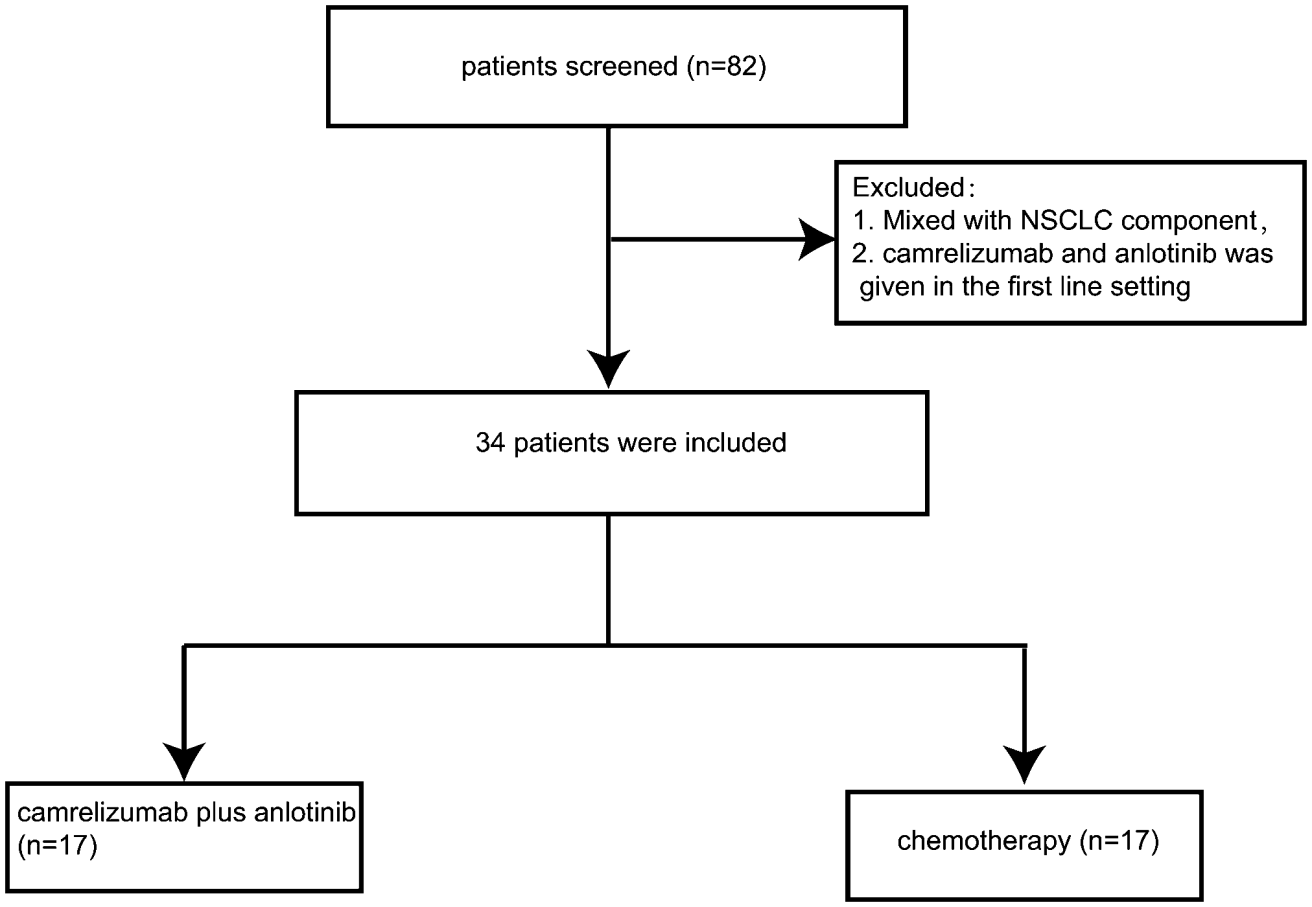
Flowchart of the study.

Out of the 82 patients reviewed, 34 met the inclusion criteria and were ultimately included in this study (**Figure 1**). These patients were divided into two treatment cohorts: the combination therapy group, comprising 17 patients treated with camrelizumab and anlotinib, and the chemotherapy group, also consisting of 17 patients who received chemotherapy (**Figure 1**). All participants had exhibited disease progression following their first-line treatment. Upon comparing clinicopathological characteristics, a general balance was observed between the two groups, although slight discrepancies were noted in the number of patients who had received radiation therapy and the proportion of patients with a history of smoking, as detailed in **Supplementary Table 1**. The median follow-up duration for this study was 32.5 months, with a 95% confidence interval (CI) ranging from 8.4 to 56.5 months.

### 3.2 Efficacy

**Figure 2:**
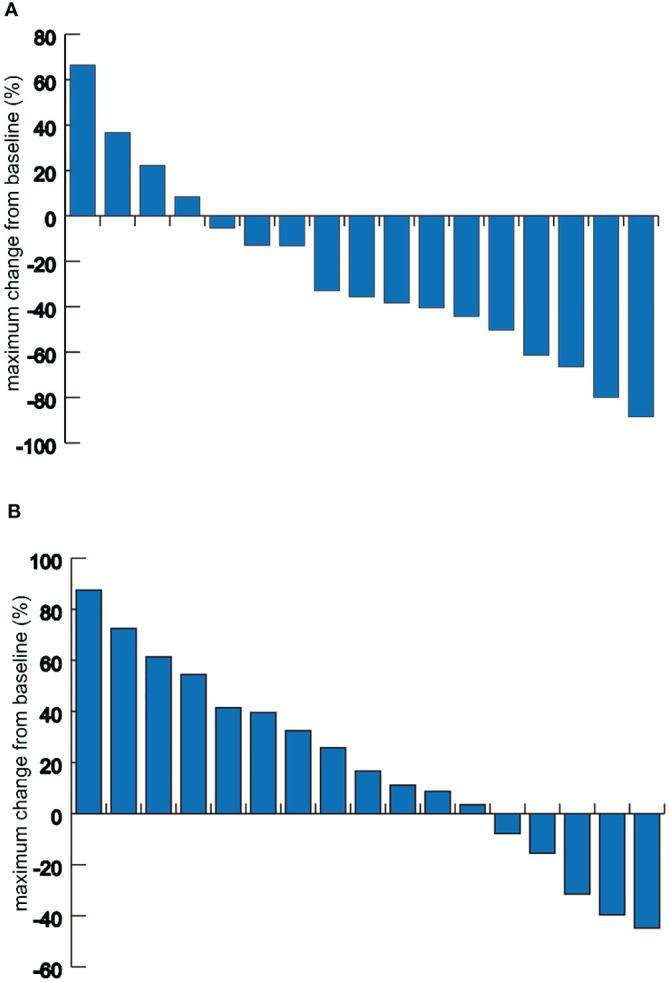
Waterfall plots of the maximum target lesion change. **(A)** Maximum target lesion change from baseline in patients of the combination therapy group. **(B)** Maximum target lesion change from baseline in patients of the chemotherapy group.

In the combination therapy group, 9 out of 17 patients (52.9%; 95% CI, 26.5-79.4%) achieved an objective response at the time of data collection. The DCR in this group was 82.4% (95% CI, 62.1-102.6%). In the chemotherapy group, the ORR was 23.5% (95% CI, 1-46%), and the DCR was 58.8% (95% CI, 32.7-84.9%) (**Supplementary Table 2**). Additionally, in the combination therapy group, 76.5% of patients (13 out of 17) demonstrated a reduction in tumor size from baseline in the target lesions (**Figure 2**). The median largest dimensional change in target lesions was -28.93% (range: -88.5% to +36.7%) for the combination therapy group, compared to 12.98% (range: -55.8% to +87.5%) for the chemotherapy group.

**Figure 3:**
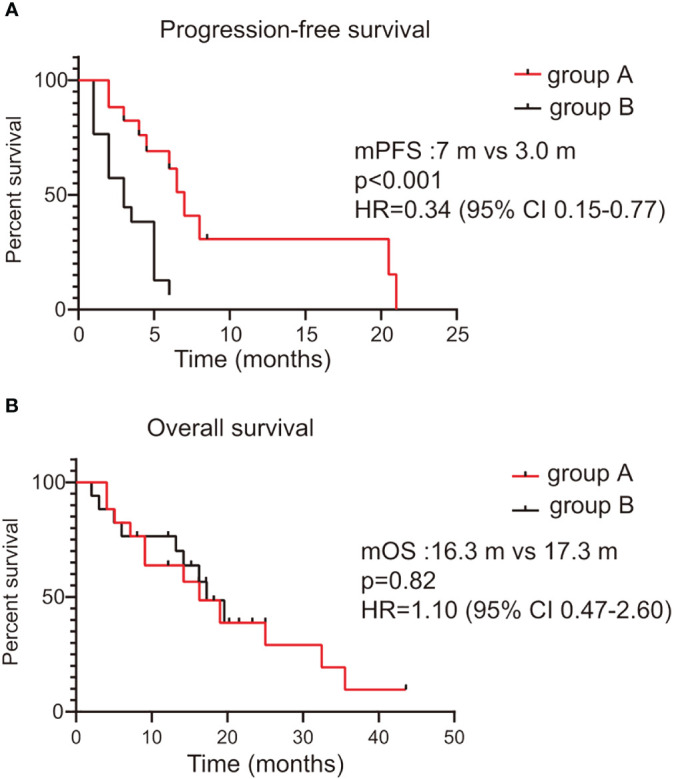
Kaplan–Meier survival curve of progression-free survival and overall survival between the combination therapy group (Group A) and the chemotherapy group (Group B). **(A)** Progression-free survival; **(B)** Overall survival. CI, confidence interval; HR, hazard ratio.

The median PFS was 7 months for the combination therapy group and 3 months for the chemotherapy group, reflecting a favorable hazard ratio (HR = 0.34, 95% CI, 0.15–0.77, p<0.001; **Figure 3A**). However, there was no statistically significant difference in OS between the two groups, with an OS of 16.3 months for the combination therapy group compared to 17.3 months for the chemotherapy group (p=0.82, **Figure 3B**).

**Figure 4:**
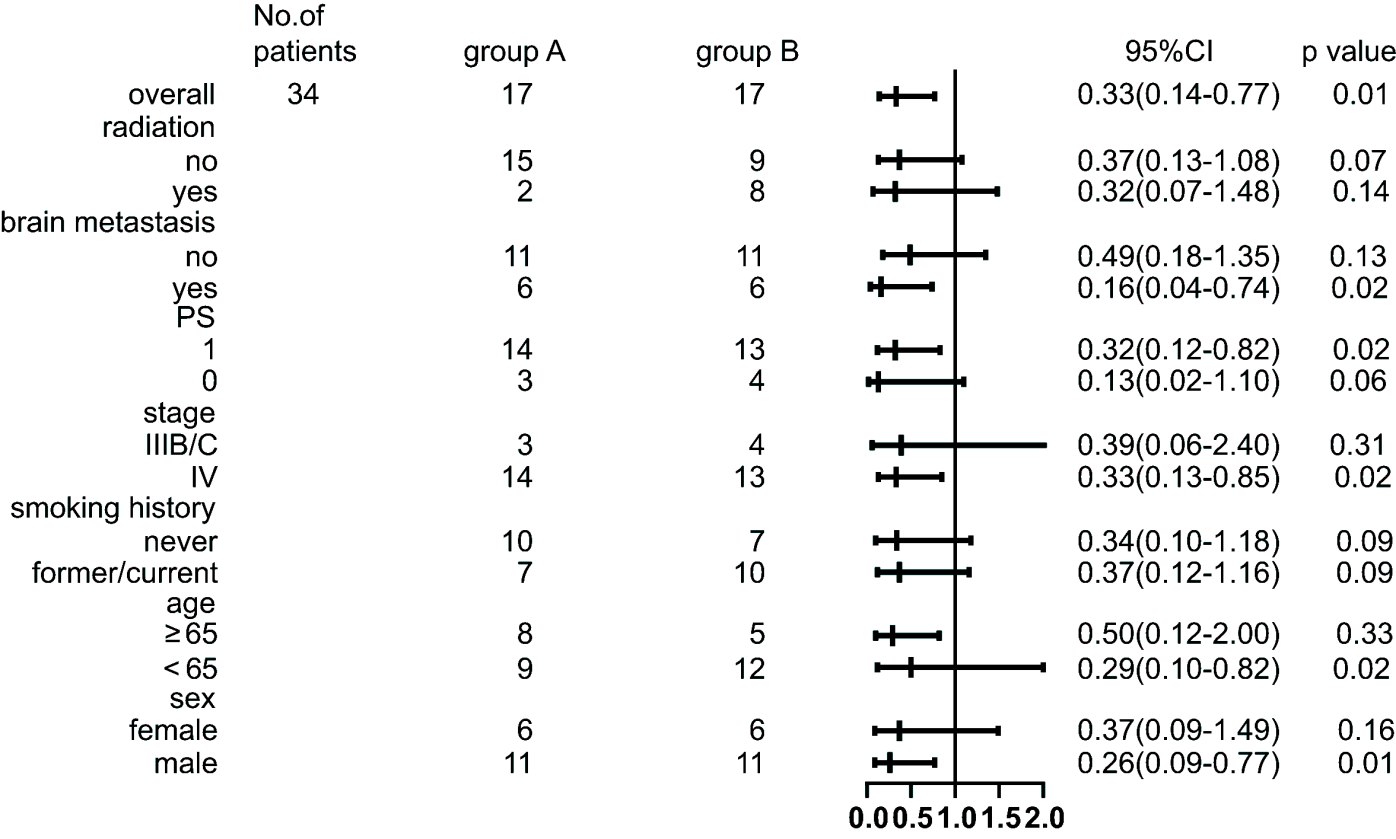
Subgroup analyses of progression-free survival.

Univariate analysis revealed that patients with an ECOG PS of 0 experienced better PFS compared to those with a ECOG PS score of 1 (HR=0.41, 95% CI, 0.18-0.94, p=0.036; **Table 1**). Furthermore, multivariate Cox regression models incorporating age, sex, ECOG PS score, presence of brain metastases, smoking history, and history of radiation treatment demonstrated that both being in the combination therapy group (HR=0.34, 95% CI, 0.15-0.75, p=0.008) and having a ECOG PS score of 0 (HR=0.34, 95% CI, 0.13–0.86, p=0.023) were significantly associated with improved PFS (**Table 1**). Subgroup analyses also indicated a significant advantage for the combination therapy group in terms of PFS across most subgroups (**Figure 4**).

**Table 1:** Univariable and multivariable analysis of progression-free survival.

Characteristics	Univariable analysis			Multivariable analysis		
	HR	95%CI	*p*	HR	95%CI	*p*
Age (≥65 vs< 65)	0.67	0.31-1.44	0.30			
Sex (Male vs female)	1.34	0.61-2.91	0.46			
Smoking status (Never vs former/current	0.80	0.37-1.71	0.56			
ECOG PS (0 vs 1)	0.41	0.18-0.94	0.036	0.34	0.13-0.86	0.023
Stage (IV vs IIIB/C)	0.47	0.15-1.41	0.18			
Brain metastasis (Yes vs no)	0.83	0.38-1.83	0.66			
Radiation (Yes vs no)	1.4	0.6-3.46	0.414			
Treatment group (Combination therapy group vs chemotherapy group)	0.33	0.14-0.77	0.01	0.34	0.15-0.75	0.008

ECOG PS, Eastern Cooperative Oncology Group performance status; HR, hazard ratio; CI, confidence interval.

### 3.3 Safety

Treatment-related adverse events (TRAEs) were observed in 14 out of 17 patients (82.4%) in the combination therapy group and in 15 out of 17 patients (88.2%) in the chemotherapy group (**Supplementary Table 3**). Within the combination therapy group, grade 3 or higher TRAEs occurred in three patients (17.6%), including one patient each with elevated alanine aminotransferase or aspartate aminotransferase, pneumonitis, and hypertension. Conversely, the chemotherapy group experienced grade 3 or higher TRAEs in five patients, including two cases of neutropenia, and one instance each of vomiting, anemia, and thrombocytopenia.

Additional TRAEs in the combination therapy group included rash (5.9%), reactive cutaneous capillary endothelial proliferation (RCCEP) (5.9%), nausea (11.8%), decreased appetite (11.8%), hypothyroidism (5.9%), neutropenia (5.9%), and anemia (11.8%). Patients in the chemotherapy group commonly experienced chemotherapy-associated adverse events, which included nausea in five patients (29.4%) and reduced appetite in two patients (11.8%) (**Supplementary Table 3**).

## 4 Discussion

To the best of our knowledge, this study is the first to report that camrelizumab combined with anlotinib outperforms standard chemotherapy in the second-line treatment for patients with ES-SCLC. This finding is distinct from previous reports, such as the case report by Yuqi Jiang et al., which involved a patient with limited-stage SCLC (22). Our study specifically focuses on ES-SCLC, providing comprehensive evidence of efficacy and safety. The median PFS for the combination therapy was a notable 7 months, significantly surpassing the median PFS observed in the chemotherapy group and those reported in previous second-line treatment studies (7–9, 23). Although the differences in the ORR and DCR did not reach statistical significance, positive trends were observed. Moreover, the combined treatment regimen demonstrated a manageable safety profile with fewer grade 3 or higher TRAEs compared to chemotherapy. These outcomes suggest that the camrelizumab and anlotinib combination could be a favorable option for second-line treatment in the ES-SCLC context.

Topotecan is currently the conventionally recommended second-line treatment option for patients with ES-SCLC following initial chemotherapy. Previous research highlighted that OS associated with topotecan in the second-line setting was limited to an average of 26 weeks, alongside a modest ORR of 7% and a higher incidence of toxic effects (10). Furthermore, in a phase 3 clinical trial, SCLC patients given topotecan after failing first-line therapy reported a 16.9% ORR, with a median PFS of 3.5 months and an OS of 7.8 months (7). More recently, the approval of lurbinectedin as a new second-line agent for SCLC resulted in a median PFS of 3.5 months and an OS of 9.3 months (24). Given the suboptimal outcomes of the established second-line regimens, the need for more effective treatments is urgent. Our research showcases a promising alternative; the combination of camrelizumab with anlotinib achieved a median PFS of 7 months in our study, which exceeds the PFS seen with the current standard second-line therapies.

The introduction of PD-1/PD-L1 inhibitors has significantly transformed the therapeutic landscape for ES-SCLC. The integration of PD-L1 inhibitors with platinum-based chemotherapy has become a standard first-line treatment, substantially improving OS for ES-SCLC patients (11, 12). Similarly, for advanced NSCLC, the combination of PD-1/PD-L1 inhibitors with platinum-based chemotherapy has received approval for first-line use (25). Moreover, the IMPOWER 150 trial demonstrated that anti-PD-L1 agents, when combined with bevacizumab and platinum-based chemotherapy, can enhance the prognosis of advanced NSCLC (26). These advancements highlight the potential of PD-1/PD-L1 inhibitor combination therapies to enhance the efficacy of immunotherapy.

Combining ICIs with angiogenesis inhibitors has shown promise as a therapeutic strategy. A phase 1 clinical trial reported an ORR of 19.6% for metastatic melanoma patients treated with ipilimumab, an anti-CTLA-4 antibody, in combination with the angiogenesis inhibitor bevacizumab (27). Similarly, other studies have demonstrated encouraging ORRs in patients with renal and urothelial cancers when ICIs are combined with angiogenesis inhibitors (28, 29). Supporting these findings, research by Fan et al. demonstrated that the combination of camrelizumab, a PD-1 inhibitor, with apatinib, an angiogenesis inhibitor, significantly improved ORR and PFS in ES-SCLC patients (23). Given the generally low ORR associated with ICI monotherapies, adding an angiogenesis inhibitor could offer a viable and relatively safe combination therapy. Our results align with this approach, suggesting that the combination of the PD-1 inhibitor camrelizumab with the oral VEGFR inhibitor anlotinib achieved an ORR of 52.9% and a DCR of 82.4%. These outcomes are noteworthy compared to a recent phase 2 trial that reported a confirmed ORR of 33.9% and a DCR of 69.5% with a regimen of camrelizumab and apatinib (23). One potential reason for the superior results in our study could be the inclusion of more patients treated with anlotinib, which previous research has indicated may be more effective and better tolerated than apatinib, especially in third-line or subsequent treatment settings for ES-SCLC (30). The relatively small number of participants in our study might also have influenced the results. Furthermore, the treatment regimen analyzed here achieved a median PFS of 7 months, a significant improvement compared to the 3-month PFS observed in the chemotherapy group (p<0.001). This finding supports the notion that combining ICIs with oral VEGFR inhibitors could be a viable and effective treatment option for ES-SCLC patients who do not respond to first-line therapy.

Patients who have received extensive prior treatment often present with a lower ECOG PS, which can indicate a reduced ability to tolerate later-line treatments, particularly chemotherapy. This trend was also noted in our study, where we observed that patients receiving chemotherapy experienced a higher incidence of TRAEs of all grades, including grade 3 or above TRAEs. However, these differences did not reach statistical significance. Previous research suggests that combination therapies are generally associated with a higher frequency of adverse events compared to monotherapies (31, 32). Nevertheless, in our study, the chemotherapy group exhibited a different safety profile, influencing the comparative assessment. As a result, the combination of camrelizumab and anlotinib demonstrated a more manageable safety profile. Our data suggest that the camrelizumab and anlotinib combination may offer a more tolerable and effective treatment alternative.

It is important to acknowledge certain limitations of this study. Firstly, due to its retrospective design, the findings should be interpreted with caution. Additionally, the limited sample size may introduce selection bias. Therefore, conclusions drawn from this study require validation through larger, randomized controlled trials.

## 5 Conclusions

In summary, the results of our study suggest that the combination of camrelizumab and anlotinib offers superior efficacy and a manageable safety profile compared to conventional systemic therapy for patients with ES-SCLC in a second-line treatment setting. Our research provides valuable insights for the treatment of ES-SCLC patients failing first-line therapy. Nonetheless, future research is necessary to further corroborate these findings.

## Data availability statement

The original contributions presented in the study are included in the article/**Supplementary Material**. Further inquiries can be directed to the corresponding authors.

## Ethics statement

The studies involving humans were approved by the Ethics Committee of the First Affiliated Hospital of Zhengzhou University. The studies were conducted in accordance with the local legislation and institutional requirements. The ethics committee/institutional review board waived the requirement of written informed consent for participation from the participants or the participants’ legal guardians/next of kin because of the retrospective design and individuals information will not be published, hence informed consent was exempted.

## Author contributions

SS: Data curation, Formal analysis, Methodology, Project administration, Resources, Writing – original draft, Writing – review & editing. XL: Conceptualization, Formal analysis, Investigation, Methodology, Project administration, Resources, Software, Writing – original draft, Writing – review & editing. SG: Methodology, Project administration, Resources, Writing – original draft, Writing – review & editing. LX: Conceptualization, Data curation, Formal analysis, Project administration, Supervision, Validation, Visualization, Writing – original draft, Writing – review & editing. NY: Conceptualization, Data curation, Funding acquisition, Investigation, Methodology, Project administration, Resources, Writing – original draft, Writing – review & editing.
