# Supplementary material for: Camrelizumab combined with anlotinib as second-line therapy for metastatic or recurrent small cell lung cancer: a retrospective cohort study
Source: Front Oncol. 2024 Jul 8;14:1391828. doi: 10.3389/fonc.2024.1391828 (PMC11261159; doi:10.3389/fonc.2024.1391828)
Supplement: Supplementary file 1 [file Table_1.docx]

Supplemental Table 1 Patient demographics and characteristics

| Characteristics | Group A (n=17) | Group B (n=17) | *p*-value |
| --- | --- | --- | --- |
| Median age(range) | 60 (21-79) | 56 (32-71) | 0.32 |
| Sex, n (%)  Male  Female | 11 (64.7)  6 (35.3) | 11 (64.7)  6 (35.3) | 1.0 |
| Smoking status  Never  Former/current | 10 (58.8)  7 (41.2) | 7 (41.2)  10 (58.8) | 0.30 |
| PS  0  1 | 3 (17.6)  14 (82.4) | 4 (23.5)  13 (76.5) | 0.67 |
| Stage  IIIB/IIIC  IV | 3 (17.6)  14 (82.4) | 4 (23.5)  13 (76.5) | 0.67 |
| Radiation  Yes  No | 2 (11.8)  15 (88.2) | 8 (47.1)  9 (52.9) | 0.024 |
| Brain metastasis  Yes  No | 11 (64.7)  6 (35.3) | 11 (64.7)  6 (35.3) | 1.0 |

Supplemental table 2 Tumour response

| response | Group A (n=17) | Group B (n=17) | *P* value |
| --- | --- | --- | --- |
| ORR | 9 (52.9%;95% CI: -26.5%-79.4%) | 4 (23.5%; 95%CI, 1%-46%) | 0.08 |
| DCR | 14 (82.4%;95% CI 62.1%-102.6%) | 10(58.8%; 95%CI, 32.7%-84.9%) | 0.26 |
| CR | 0 | 0 |  |
| PR | 9 (52.9%) | 4 (23.5%) |  |
| SD | 5 (29.4%) | 6 (35.3%) |  |
| PD | 3 (17.6%) | 7 (41.2%) |  |

Supplemental Table 3 Adverse events

|  | Group A (n=17) | | Group B (n=17) | |
| --- | --- | --- | --- | --- |
| TRAEs | All grades | Grade ≥3 | All grades | Grade ≥3 |
| Any | 14 (82.4%) | 3 (17.6%) | 15 (88.2%) | 5 (29.4%) |
| Rash | 1 (5.9%) | 0 | 0 | 0 |
| RCCEP | 1 (5.9%) | 0 | 0 | 0 |
| Nausea | 2 (11.8%) | 0 | 5 (29.4%) | 0 |
| Appetite decreases | 2 (11.8%) | 0 | 2 (11.8%) | 0 |
| Vomiting | 0 | 0 | 3 (17.6%) | 1 (5.9%) |
| ALT/AST increase | 3 (17.6%) | 1 (5.9%) | 0 | 0 |
| Anemia | 2 (11.8%) | 0 | 2 (11.8%) | 1 (5.9%) |
| Neutropenia | 1 (5.9%) | 0 | 2 (11.8%) | 2 (11.8%) |
| Thrombocytopenia | 0 | 0 | 1 (5.9%) | 1 (5.9%) |
| Hypothyroidism | 2 (11.8%) | 0 | 0 | 0 |
| Pneumonitis | 1 (5.9%) | 1 (5.9%) | 0 | 0 |
| Hypertension | 1 (5.9%) | 1 (5.9%) |  |  |

VEGF/VEGFR: vascular endothelial growth factor/vascular endothelial growth factor receptor, TRAEs: treatment related adverse events, RCCEP: reactive cutaneous capillary endothelial proliferation, ALT/AST: alanine aminotransferase/aspartate aminotransferase
